# Differential regulation of the *foraging *gene associated with task behaviors in harvester ants

**DOI:** 10.1186/1472-6785-11-19

**Published:** 2011-08-10

**Authors:** Krista K Ingram, Lindsay Kleeman, Swetha Peteru

**Affiliations:** 1 Department of Biology, 13 Oak Drive, Colgate University, Hamilton, NY 13346, USA

## Abstract

**Background:**

The division of labor in social insect colonies involves transitions by workers from one task to another and is critical to the organization and ecological success of colonies. The differential regulation of genetic pathways is likely to be a key mechanism involved in plasticity of social insect task behavior. One of the few pathways implicated in social organization involves the cGMP-activated protein kinase gene, *foraging*, a gene associated with *foraging* behavior in social insect species. The association of the *foraging *gene with behavior is conserved across diverse species, but the observed expression patterns and proposed functions of this gene vary across taxa. We compared the protein sequence of *foraging *across social insects and explored whether the differential regulation of this gene is associated with task behaviors in the harvester ant, *Pogonomyrmex occidentalis*.

**Results:**

Phylogenetic analysis of the coding region of the *foraging *gene reveals considerable conservation in protein sequence across insects, particularly among hymenopteran species. The absence of amino acid variation in key active and binding sites suggests that differences in behaviors associated with this gene among species may be the result of changes in gene expression rather than gene divergence. Using real time qPCR analyses with a harvester ant ortholog to *foraging *(*Pofor*), we found that the brains of harvester ant foragers have a daily fluctuation in expression of *foraging *with mRNA levels peaking at midday. In contrast, young workers inside the nest have low levels of *Pofor *mRNA with no evidence of daily fluctuations in expression. As a result, the association of *foraging *expression with task behavior within a species changes depending on the time of day the individuals are sampled.

**Conclusions:**

The amino acid protein sequence of *foraging *is highly conserved across social insects. Differences in foraging behaviors associated with this gene among social insect species are likely due to differences in gene regulation rather than evolutionary changes in the encoded protein. The task-specific expression patterns of *foraging *are consistent with the task-specific circadian rhythms observed in harvester ants. Whether the molecular clock plays a role in regulating *foraging *gene expression (or vice versa) remains to be determined. Our results represent the first time series analysis of *foraging *gene expression and underscore the importance of assaying time-related expression differences in behavioral studies. Understanding how this gene is regulated within species is critical to explaining the mechanism by which *foraging *influences behavior.

## Background

Recent advances in sociogenomics allow for analyses of the molecular mechanisms regulating behavioral flexibility [1-3]. In eusocial insects, one key aspect of their sociality, the division of labor, has received the most attention [4,5]. Species exhibiting age-related polyethism, a derived form of division of labor in ants and bees where colony tasks are allocated among distinct behavioral phenotypes of identical genotype, are ideal candidates for studying the molecular basis of behavioral plasticity [4,5]. The colony organization of advanced eusocial insects evolved independently in ants, bees, and wasps but little is known about the genetic mechanisms that mediate behavioral plasticity in these species.

The *foraging *gene, a cGMP-activated protein kinase gene (PKG), has emerged as an important behavioral gene in diverse taxa but the associations between the gene and behavior of particular species described to date vary in both mechanism and proposed function [6,7]. In mammals, PKG orthologs--*cGKI *and *cGKII *genes--play a role in nociception responses, learning and memory, and circadian rhythmicity [6,8]. The nematode ortholog, *egl-4*, is involved in aggregation responses and food-related behaviors in *C. elegans *[9]. Furthermore, differential expression of *egl-4 *influences EDTA sensitivity and chemotaxis in *Pristionchus pacificus*, a nematode that parasitizes scarab beetles [10].

The *foraging *gene has been shown to have a direct link to foraging behavior in several insect species [7,11-16]. Indeed, the function of *foraging *was initially described in the food-search behavior of *Drosphilia melanogaster *[15]. Differences in fruit fly foraging behavior are linked to alternative alleles that result in changes in abundance of *foraging *mRNA and protein kinase activity [15]. Later studies in fruit flies provided evidence for the influence of *foraging *on habituation and sucrose responsiveness, stress tolerance, olfactory and visual learning, memory and sleep patterns [17-19].

In the social insects, the *foraging *gene is implicated in the behavioral division of labor [11-13,16,20]. Honeybee foragers (*Apis mellifera*) have higher levels of expression of *foraging *than nurse bees and treatment with cGMP causes precocious foraging in young bees [7]. A similar result was found in bumblebees that have size-dependent task allocation; larger foragers had higher expression of *foraging *than smaller nurse bees [12]. However, as larger forager bumblebees age, the expression of *foraging *decreases, suggesting age-dependent effects on overall expression levels in this species. In previous work, we showed that a harvester ant ortholog (*Pbfor*) to *foraging *was associated with foraging behavior in ants, but in contrast to honeybees, the expression was lower in foragers than workers of other tasks, including brood care (nurse) workers [11]. These patterns were similar to those found for *Vespula vulgaris *wasps. Wasp foragers had lower expression of *foraging *than nurse wasps, although there was considerable variation in expression levels [16]. A study on another ant species, *Pheidole pallidula*, showed that expression of this gene is high in soldier castes, which do not forage, and low in minor workers which do engage in foraging [13].

It is not surprising that a conserved gene encoding for a protein kinase has multiple behavioral roles across unrelated taxa, but the differential expression of this gene within particular taxa suggests that *foraging *also plays a role in behavioral plasticity. To better understand the role of *foraging *in the plasticity of task behaviors important for the division of labor in eusocial insects, we explored two alternative hypotheses for why the association of the foraging gene and behavior varies across species. Sequence differences between species could alter binding site specificity or other properties of the encoded kinase and thus alter the effect or function of the foraging gene in particular taxa. An alternative, but not mutually exclusive, hypothesis is that the foraging gene is differentially regulated across species, thus resulting in diverse associations between *foraging *gene expression and behavior. We examined the evolution of the *foraging *gene in Hymenoptera and explored whether the differential regulation of this gene is associated with task behaviors in the harvester ant, *Pogonomyrmex occidentalis*.

Red harvester ants in the genus *Pogonomyrmex *live in large colonies of up to 10-12,000 workers in the southwestern deserts of the United States [21]. All workers in a colony are similar morphologically, but on a given day, some individuals forage for seeds, while other individuals perform other colony tasks [21-23]. In ants, younger workers tend to remain inside the nest while older workers perform tasks outside, such as foraging [24]. In harvester ants, this progression of worker tasks, known as age-related polyethism, occurs over the course of a year, the approximate lifespan of a worker [25,26]. In harvester ants, young workers perform tasks related to brood care and never (or rarely) leave the nest. Workers then progress to nest maintenance tasks, with brief trips out of the nest, then to patrolling tasks, with short morning forays from the nest, and finally to foraging tasks [21,23,27-29]. Foragers spend the most time out of nest, leaving in early morning and foraging until mid-afternoon.

The recent discovery of task-specific expression of circadian clock genes in harvester ants confirmed that foragers have a functional molecular clock and circadian rhythm, but workers inside the nest do not show pronounced circadian rhythms in activity levels or expression of clock genes [30]. In conjunction with the large differences in behavioral repertoire between tasks (with forager behaviors tightly linked to time of day), these results suggest that other behavioral genes may be differentially regulated via downstream interactions with molecular clock pathways. Here, we show that the expression pattern of the *foraging *gene changes during the course of a day in harvester ant foragers. In addition, nest workers that stay inside the nest with no evidence of active circadian rhythm have low levels of *foraging *mRNA and little fluctuation in mRNA levels throughout the day. The fact that *foraging *mRNA levels vary with time of day is of critical importance to studies attempting to link the expression of the *foraging *gene with behavioral function.

## Results

### Gene evolution in *foraging*

**Figure 1:**
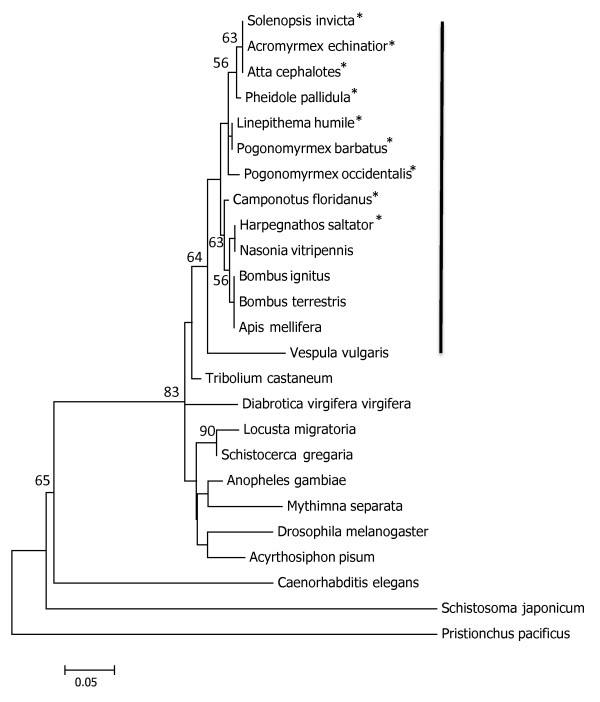
**Maximum likelihood analysis of *foraging *amino acid sequence**. Maximum likelihood analysis of amino acid sequences of *foraging *from insect taxa with *C. elegans, S. japonicum*, and *P. pacificus *as outgroups. The tree shown represents the bootstrap consensus tree of 1000 replicates. Branch lengths represent the number of substitutions per site. All positions containing gaps and missing data were eliminated for a total of 367 positions in the final dataset. The black bar denotes the hymenopteran species, the non-social species are shown in bold type, and the ants (Formicidae) marked with an asterisk.

Comparative analysis of amino acid sequence from the open reading frame of *foraging *shows considerable conservation of this gene among ants and maximum likelihood phylogenetic analysis shows short branch lengths between ant species and other eusocial insects (Figure 1). For example, *Harpegnathos saltator*, the Indian jumping ant, and *Camponotus floridanus*, a carpenter ant, group with *Nasonia vitripennis*, a non-social wasp in an unresolved cluster of other hymenopteran species (BP = 63). Within ants, amino acids sequences have ClustalW similarity scores of 96.4, with an average of approximately 4% of the amino acid sites differing between pairs of sequences. In comparison, the similarity score across other eusocial insect species is 87.5. Average similarity scores between ants and other species reflect phylogenetic expectations (other eusocial insects: 90.4, Nasonia (non-social Hymenopteran): 89.7, non-Hymenopteran insects: 83.6, Nematodes/Trematodes: 52).

**Figure 2:**
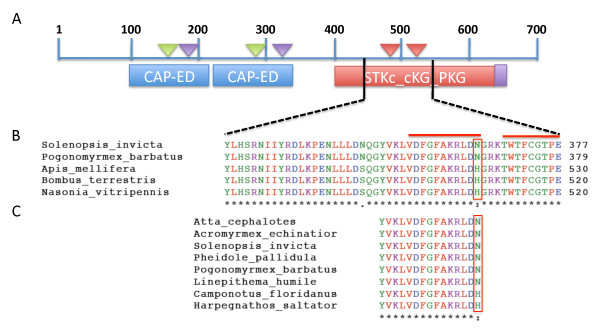
**Annotation of the conserved domains of the *foraging *gene**. A) Annotation of the cGMP-dependent protein kinase *foraging *showing conserved domains. CAP-ED: effector domain of the CAP family of transcription factors, STKc_cGK_PKG: catalytic domain of the cGMP-dependent protein kinase. B) Partial sequence alignment of catalytic domain in hymenoptera showing the single amino acid replacement in the A-loop region (asparagine/histidine). The red line indicates the two A-loop regions and the red box encloses the amino acid replacement. C) Partial sequence alignment of A-loop region in catalytic domain in known Formicidae. The red box encloses the asparagine/histidine replacement site in eight ant species. The asparagine mutation is present in 6 out of 8 species.

The *foraging *gene is a cGMP-dependent kinase (cGPK) that contains four conserved domains; two tandem effector domains of the CAP family of transcription factors (CAP-ED), a catalytic domain of the protein Serine/Threonine kinase, and a protein kinase C terminal domain (Figure 2A). Within the Hymenoptera, there are 50 amino acid changes across the gene, with 20 changes occurring within the conserved domains (Additional File 1). There are only 4 amino acid replacements unique to ants within the conserved domains. No changes occurred at ligand-binding sites or flexible hinges in the CAP-ED domains and no changes occurred at the ATP binding sites or actives sites of the PKG catalytic domain with the exception of a single amino acid change in the activation loop (A-loop). In most Hymenoptera, the final amino acid in the A-loop chain is a histidine but some ants have an asparagine (Figure 2B). Both amino acids have polar side chains but the difference in side chain conformation may alter the activation efficiency or represent an additional function of the enzyme. As this amino acid change is not conserved across all ant species (Figure 2C), it is not a change that is likely to account for the alternative expression patterns of the *foraging *gene across the Hymenoptera.

### Differential expression of *foraging *between tasks

**Figure 3:**
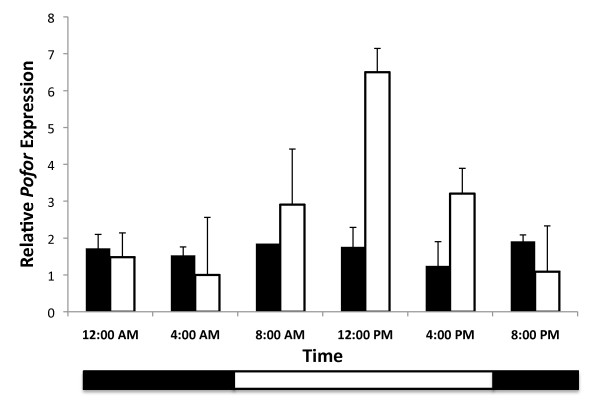
**Relative expression of foraging mRNA in workers over time**. Relative expression of *foraging *mRNA in individual worker brains from experimental colonies of *P. occidentalis*. Colonies were entrained in controlled environmental chambers in a 12:12 hr LD regime. Foragers (light bars, n = 4 colonies) and nest workers (dark bars, n = 4 colonies) were collected at six time points. Bars represent the averages of colony means with three nest workers and three foragers sampled per colony (SE calculated across colonies). The open stripe in the horizontal bar at base of the plot represents the light phase and the solid stripe represents the dark phase.

The results of the mixed model repeated measures ANOVA show gene expression varies significantly across time points (F = 5.33, p < 0.001). As predicted, this change in gene expression across time was significantly different across tasks (Figure 3; F = 2.97, p < 0.05). The pattern of gene expression over time also varied with colony (F = 3.48, p < 0.001) and with a colony × task interaction (F = 11.21, p < 0.001). Collapsing across time, none of the between subjects factors or their interactions were significant (Additional File 2).

Over all time points, the average expression level of *foraging *is 2.4 times greater in foragers than nest workers, but due to variation across time points, this difference is not significant (t = 1.75; p = 0.08). The highest mRNA levels for foragers are recorded at midday. At this time, foragers have more than three times the level of *foraging *mRNA than nest workers (t = 2.56, p = 0.05). During late evening and early morning hours, when forager levels dip to the lowest level, nest workers have marginally higher average levels of *foraging *mRNA than foragers (t = 1.98, p = 0.06).

The pattern of expression of *Pofor *mRNA in forager brains is significantly and strongly correlated with the generalized sine function curve predicted from the daily fluctuations in foraging behavior (t = 4.13, r = 0.92, p = 0.01). The expression pattern of nest workers is not correlated with the predicted sine curve function (t = 0.30, r = 0.17, p = 0.39). Comparing the two tasks, foragers show a significantly higher correlation to the predicted daily fluctuation in expression levels of *foraging *than nest workers (t = 2.72, r = 0.74, p = 0.03).

## Discussion

Molecular mechanisms driving differences in social insect behavior can involve gene evolution--mutations in protein sequence of behavioral genes--or changes in gene regulation--mutations in non-coding regions that ultimately affect the impact of such genes on the behavior of organisms. Our results show that an important behavioral gene, *foraging*, is highly conserved in the Hymenoptera, with little evidence for functional evolution in amino acid sequence. Furthermore, our results demonstrate that differential regulation of this gene in *P. occidentalis *is associated with task-specific behaviors that are integral to the division of labor in a social insect. The regulation of *foraging *in harvester ants is not limited to differences in levels of *foraging *mRNA but involves daily fluctuations in gene expression in foragers that are not seen in workers inside the nest. Thus, differential regulation of *foraging *gene expression may play an important role in both influencing species-specific behavior and regulating behavioral plasticity within species.

### Conservation in *foraging *gene coding sequence

Our results indicate that both the protein sequence of *foraging *and the association of the *foraging *gene with food-related behavior are conserved across hymenopteran species. Thus, the most likely mechanism by which *foraging *influences behavior within species appears to operate primarily via differential regulation of gene expression. Changes in the pathways responsible for the regulation of *foraging*, rather than *foraging *protein sequences, are also likely to be responsible for the variability in the expression patterns of the this gene across species. Although the link between the *foraging *gene and foraging behavior is conserved across eusocial insects, the directionality of the relationship may differ between species due to independent mutations in the regulatory pathways [2]. Thus, there are potentially two levels at which the differential regulation of *foraging *may influence flexibility in social insect division of labor; age-related or task-specific plasticity in worker behavior within species and species-dependent associations of *foraging *gene expression and worker behavior.

There is a growing body of evidence that additional genetic pathways involved in nutrition and metabolism also play a major role in worker-worker division of labor (reviewed in [4]). Causal relationships between gene regulation and transitions in worker task behaviors have been documented for a few of these genes, including *foraging *[7,14]. The results from these studies highlight the complexity of relationships between conserved genetic pathways and transitions to foraging in social insects. For example, *malvolio*, a gene involved in manganese transfer and sucrose responsiveness, has a higher expression level in pollen foragers than nurse honeybees with nectar foragers intermediate [31]. Manganese treatment causes precocious foraging--but does not increase pollen foraging in honeybee colonies. RNAi knockdown of the storage protein gene, *vitellogenin *(*Vg*) also causes precocious foraging in honeybees among other pleiotropic effects, demonstrating the co-option of a conserved reproductive regulatory pathway for the organization of behavior [32]. The insulin-signaling TOR pathway is an important metabolic pathway linked to foraging behavior in honeybees and *Polistes *wasps [33,34]. IIS-gene expression is up-regulated in forager bees which have diminished nutrient reserves relative to nurse bees--a pattern opposite to expectations from caste differentiation and associations of IIS with nutrient status in other insect species [35]. Interesting parallels can be drawn between the IIS pathway and the *foraging *pathway. In both cases, differential regulation occurs within and between species, suggesting evolutionary changes in regulatory regions influencing these pathways. Understanding the diversity of mechanisms by which conserved molecular pathways regulate behavioral plasticity in workers is a central issue in social insect biology. The *foraging *gene provides an important example of how differential regulation of conserved genes influences the behavioral division of labor within and between social insect species.

### Behavioral plasticity and *foraging *gene regulation

In harvester ants, the *foraging *gene is expressed in the brains of both foragers and workers inside the nest, but the pattern of expression differs between the two tasks during the course of the day. Foragers have higher expression levels of *foraging *during midday when the foragers are outside the nest, actively foraging. In contrast, the expression levels of nest workers do not vary with time of day, a pattern consistent with the round-the-clock locomotor activity patterns of these workers [30]. Nest workers generally show lower levels of *Pofor *mRNA than foragers during the day and similar, or higher, levels in late evening and early morning hours.

A previous study on a related harvester ant, *Pogonomyrmes barbatus*, analyzed task-specific *foraging *expression in five different tasks, including young workers that tended brood inside the nest and foragers from field colonies [11]. The results from that study showed that foragers had a lower expression of *PbFor *than brood workers [11]. Interestingly, the sampled workers from the *P. barbatus *field study were collected in the early morning (between 6-8 am--a convention that allowed researchers to identify and sample the workers of all tasks in the field). The results from the *P. barbatus *study support the findings from the current study, with average levels of *Pofor *mRNA higher in nest workers than foragers in the early morning. However, the conclusions from the 2005 study were limited to a single time of the day. In order to clarify the role of *foraging *in field colonies of harvester ants, it will be important to test whether *P. barbatus *foragers show an increase in expression of *foraging *throughout the day as seen in lab colonies of *P. occidentalis *foragers. Alternatively, it is possible that *P. barbatus *foragers in field colonies show an age-related decrease in *foraging *expression similar to the decrease documented in bumblebees [12]. In laboratory colonies, foragers may be newly transitioned from nest work and may not show a decrease in *foraging *expression due to age. The high level of *Pofor *mRNA in *P.occidentalis *foragers in the laboratory may represent the increase in *foraging *expression associated with new external stimuli and the rapid learning associated with foraging behavior [6]. Further data from field and laboratory studies in both species is needed to help disentangle these two hypotheses.

Our results also highlight the importance of considering the effect of time and circadian rhythms on gene expression and, in particular, the expression of important behavioral genes. Despite the cost of designing extensive qPCR studies, data we present indicate that it may be necessary to use multiple time points when sampling behavioral gene expression differences within and between species. At this point, we understand few of the molecular pathways affected by circadian circuitry, although a number of recent studies are attempting to narrow this gap in our knowledge [6,36]. It is important to note if task-related variation in *foraging *expression levels across species is due to mutations in regulatory mechanisms, then even closely related species may exhibit variation in *foraging *expression patterns. The extreme flexibility in the regulation of *foraging *expression underscores the potential importance of this gene in the development of behavioral plasticity in social insect workers. Alternatively, our results also highlight the possibility that some of the experimental differences in patterns of *foraging *expression reported across studies may not represent distinct associations of *foraging *gene expression with species-specific behavior, but rather differences in the timing of sample collections and the inadvertent capture of discrete snapshots of expression levels across the daily fluctuations of the gene. A review of the methods in previous social insect studies did not provide enough detail on the timing of sampling to determine whether this may be a factor affecting different patterns of expression across species.

The fact that we found significant levels of expression of the *foraging *gene in nest workers suggests that this gene has more than one function in harvester ant behavior. The possibility of multiple functions for *foraging *within a species is not entirely surprising as the *foraging *gene encodes for a protein kinase that is expected to have numerous downstream targets [8]. There is already evidence for numerous functions of the *foraging *gene in behavior including phototaxis, chemotaxis, foraging, learning and memory [6,8]. In harvester ants, task-specific behavioral associations with the *foraging *gene are likely to be dependent on development due to the age differences between task. The relatively stable expression in young workers that remain inside the nests may indicate a specific, novel function for this gene that does not involve phototaxis or foraging activities outside the nest. We recognize that gene expression and the presence of *foraging *mRNA do not necessarily transfer to protein activity differences *in vivo*. Important future studies could test for expression patterns in FOR protein in specific brain regions in foragers versus nest workers to elucidate whether the differential regulation of *foraging *is limited to particular brain areas in different tasks or during different stages of development. A recent study on another ant *Pheidole pallidula *showed the tremendous promise of research in this area [13].

## Conclusions

The *foraging *gene is highly conserved across eusocial species and has a robust association with task-specific behaviors involved in the division of labor within colonies. Differences in how *foraging *influences task behaviors across social insects appear to be a product of gene regulation, rather than protein evolution. In laboratory colonies of harvester ants, the regulation of the *foraging *gene shows task-dependent daily fluctuations in *Pofor *mRNA that correlate with patterns of foraging behavior. The functions of *foraging *are not yet known for ants but it is clear that the flexibility in the regulation of this gene is associated with the plasticity in worker behavior within species and is likely to play an important role in the evolution of behavioral differences between species.

## Methods

### Ants and sampling

Laboratory colonies of harvester ants (*Pogonomyrmex occidentalis*) were established by collecting field colonies from Hurricane, Utah. Colonies were transferred to individual plexiglass nest boxes (15 cm wide × 30 cm long × 10 cm high) connected to open-air foraging arenas (25 cm W × 60 cm L × 15 cm H) by short Tygon tubing (2 cm diameter × 10 cm long). Nest box floors had a 3 cm layer of plaster with built-in irrigation system to keep colonies moist. Nest boxes were covered in double layers of red cellophane and had removable lids for ease of observation and collection. Colonies were kept under stable conditions of light, temperature and humidity in controlled environmental chambers (lights on/off at 6 AM/6PM; ~70% relative humidity and a constant temperature of 19°C). Laboratory colonies (n = 4) were composed of at least 400 workers and contained some larvae, but no queens.

Foragers were identified as ants that were observed on the food in the foraging arena and were labeled using a dot of acrylic paint during the day. Marking was done at least 3 days before any collection for all ants. Nests were observed for at least a week before any marking and nearly two weeks before any collections occurred. The hours of observation varied across weeks, with approximately 4 hours of observation per day occurring across peak foraging times during the day. Nest workers were identified as ants that remained in the covered nest box with brood and were never observed in the foraging arena. When possible, unmarked ants that were physically tending brood were collected as 'nest worker' samples. Exact ages of the foragers and nest workers were not known, but workers within the nest are typically younger than foragers [27,29].

We collected multiple ants (6-10) at each time point and sampled three individuals per time point per colony for RNA extraction. All sampling during evening hours was done using dim red light in dark conditions. Ants were collected from each colony at six time points: 4:00, 8:00, 12:00, 16:00, 20:00, and 24:00 hours. Individual ants were immediately immersed in liquid nitrogen and stored at -70°C until dissection. Brain dissections were performed in 50 μL 1 × PBS and 5 μL RNAlater^® ^under a dissecting microscope. RNA isolation of individual brains was done using the RNeasy Plus Mini Kit according to manufacturer's instructions (Qiagen) with an additional DNase treatment (Qiagen). Isolated RNA was quantified using Nanodrop and stored at -70°C. In this study, RNA was purified from 144 individual brain dissections, 432 cDNA reactions were amplified and 864 qPCR reactions were analyzed for the two genes.

### Phylogenetic analysis of *foraging*

Degenerate primers were designed to amplify and sequence the *Pogonomyrmex occidentalis *ortholog to *foraging *from genomic DNA and cDNA (ABI Big-Dye Sequencing technology on an ABI 377 instrument). Amino acid sequences from the open reading frame, including cGMP-binding and kinase domains and the 3' end of *Pofor*, were aligned to orthologs found in Genbank and the ant genome database http://www.antgenomics.org/ant-genomics-resources using CLUSTALW in MEGA 5.0 Additional Files 3 &4). A phylogeny was constructed using maximum likelihood methods based on the JTT matrix-based model in MEGA 5.0 with 367 informative sites. The robustness of the unrooted tree was assessed using bootstraps (1000 replicates). A maximum parsimony tree was also constructed for comparison using TNT (Additional File 5). Amino acid similarity of *Pofor *was calculated for each ortholog from the sequence alignments. Sequence features were annotated manually using the honeybee sequence as a reference (NP_001011581.1).

### Quantitative real-time PCR

Harvester ant-specific primers were designed from exon-coding regions to amplify a 130 bp region for qPCR analyses (JN255751). cDNA was synthesized from extracted total RNA preps using ABI TaqMan Gold Reverse Transcriptase reagents and and random hexamers. The 10 μL reactions included 1.2 μL of RNA with 1 × TaqMan RT Buffer, 5.5 mM 25 mM MgCl_2_, 500 μM of each of the deoxyNTPs, 2.5 μM of the Random Hexamer primers, 0.4 U/uL of RNase Inhibitor and 1.5 U/uL of MultiScribe Reverse Transcriptase (50 U/uL). Reactions were performed in triplicate for each individual brain. All reactions were run at 25°C for 10 minutes, 48°C for 30 minutes, 95°C for 5 minutes, and then stored at -20°C until quantitative PCR. For each cDNA replicate, expression of *Pofor *was assayed on an ABI 7900 HT instrument using ABI Taqman Gold reagents with the following primers: PbForB Forward: TGGTGGTGACCCAATGAAGACGTA, PbForB Reverse: TAATCCCGCGGAACGTCTTG, PbForB Probe: TCCATCACGCGTAACGCAATGGCT. The 25 uL qPCR reactions included 3 uL of template cDNA with 1 × TaqMan Buffer A, 5.5 mM 25 mM MgCl_2_, 200 μM of 10 mM deoxyATP, 200 μM of 10 mM deoxyCTP, 200 μM of 10 mM deoxyGTP, 400 μM of 20 mM deoxyUTP, 100 nM of probe, 200 nM of each primer, 0.01 U/uL of AmpErase UNG and 0.025 U/uL of AmpliTaq Gold DNA Polymerase (50 U/uL). To standardize *foraging *expression, an ant homolog of the RNA polymerase II 512kD (*PbRPII) *subunit was used as a control [37] for each cDNA replicate (JN255750). The following primers were used for the control gene: PbRPII Forward: GAGAACCAAGTGAACAGGAT, PbRPII Reverse: TTATTGTATTCAGTCAGGGATTTC, PbRPII Probe: CAGAGCCTCCAGTCTTGTCTCGA. Real-time PCR reactions for *Pofor *and *PbRPII *were performed under the following conditions: 2 min at 50°C for one cycle, 10 min at 95°C for one cycle, 15 sec at 95°C, 1 min at 58°C, for 45 cycles. Data was analyzed using SDS 2.1 software and quantification of relative mRNA levels was calculated using the ΔΔCt method. Three individual brains were averaged to calculate a colony value per time point. Colony values were averaged at each time point for comparisons across a 24 hr period.

Differences in individual brain expression levels across time and task were tested with a mixed model repeated measures ANOVA that included colony as a random factor and time as the repeated measure. Expression differences between tasks at individual time points were analyzed with standard t-tests in SPSS. Differences in the pattern of relative *Pofor *expression over time were analyzed for each task using repeated measures contrast analyses [38]. Contrast analysis allows one to test specific, theoretically driven, a priori predictions about patterns in repeated measures data. In this case, we tested the prediction that daily fluctuations in *foraging *gene expression follow observed daily rhythms in foraging behavior. The expression patterns of both foragers and nest workers (non-foragers) were compared to a generalized sine curve function with a maximum expression at midday. This sine curve approximates the daily locomotor activity rhythms of *P. occidentalis *foragers in laboratory colonies [30]. Using the same method, one can also test whether observed values from a particular task follow a predicted pattern better than another task [38]. Differences in expression patterns between foragers and nest workers were compared to test which task had a better fit to the predicted sine curve.

## Authors' contributions

KKI designed the study, participated in molecular genetic work and statistical analyses, and drafted the manuscript. LK and SP participated in the molecular genetic work and statistical analyses. All authors read and approved the final manuscript.

## Supplementary Material

Additional file 1 **Clustal alignment of *foraging *gene across Hymenoptera**. Alignment of *foraging *gene sequences using Clustal W. The four amino acid sequences that differ across Hymenoptera are boxed. Click here for file

Additional file 2 **Results from Mixed Model Repeated Measures ANOVA**. Table of complete results from mixed model repeated measures ANOVA. Click here for file

Additional file 3 **Database accession numbers for *foraging *gene**. A list of the Genbank accession numbers for *foraging *gene sequences used in phylogenetic analyses. Click here for file

Additional file 4 **Original foraging sequence alignment in ClustalW**. Alignment of *foraging *gene sequences using Clustal W with no gaps removed. This alignment was used to measure similarity scores of amino acid seqeuences between species. Click here for file

Additional file 5 **Maximum parsimony tree of *foraging *gene**. Maximum parsimony consensus tree of *foraging *gene. The tree shown represents the bootstrap consensus tree of 250 replicates. Click here for file
