# Supplementary material for: Differential regulation of the foraging gene associated with task behaviors in harvester ants
Source: BMC Ecol. 2011 Aug 10;11:19. doi: 10.1186/1472-6785-11-19 (PMC3180247; doi:10.1186/1472-6785-11-19)
Supplement: Additional file 1 — Clustal alignment of foraging gene across Hymenoptera. Alignment of foraging gene sequences using Clustal W. The four amino acid sequences that differ across Hymenoptera are boxed. [file 1472-6785-11-19-S1.PPT]

## Slide 1
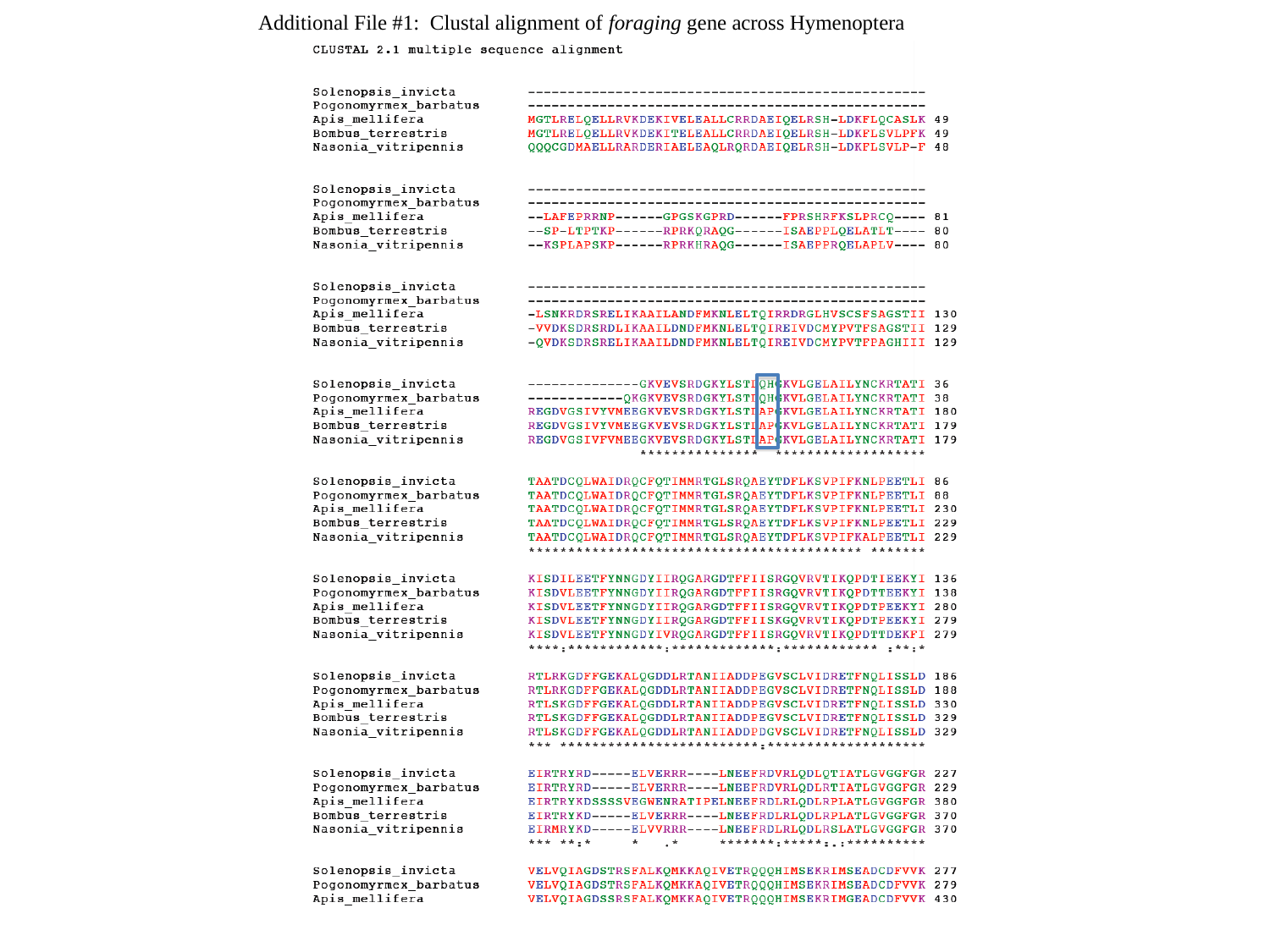

Additional File #1: Clustal alignment of foraging gene across Hymenoptera

## Slide 2
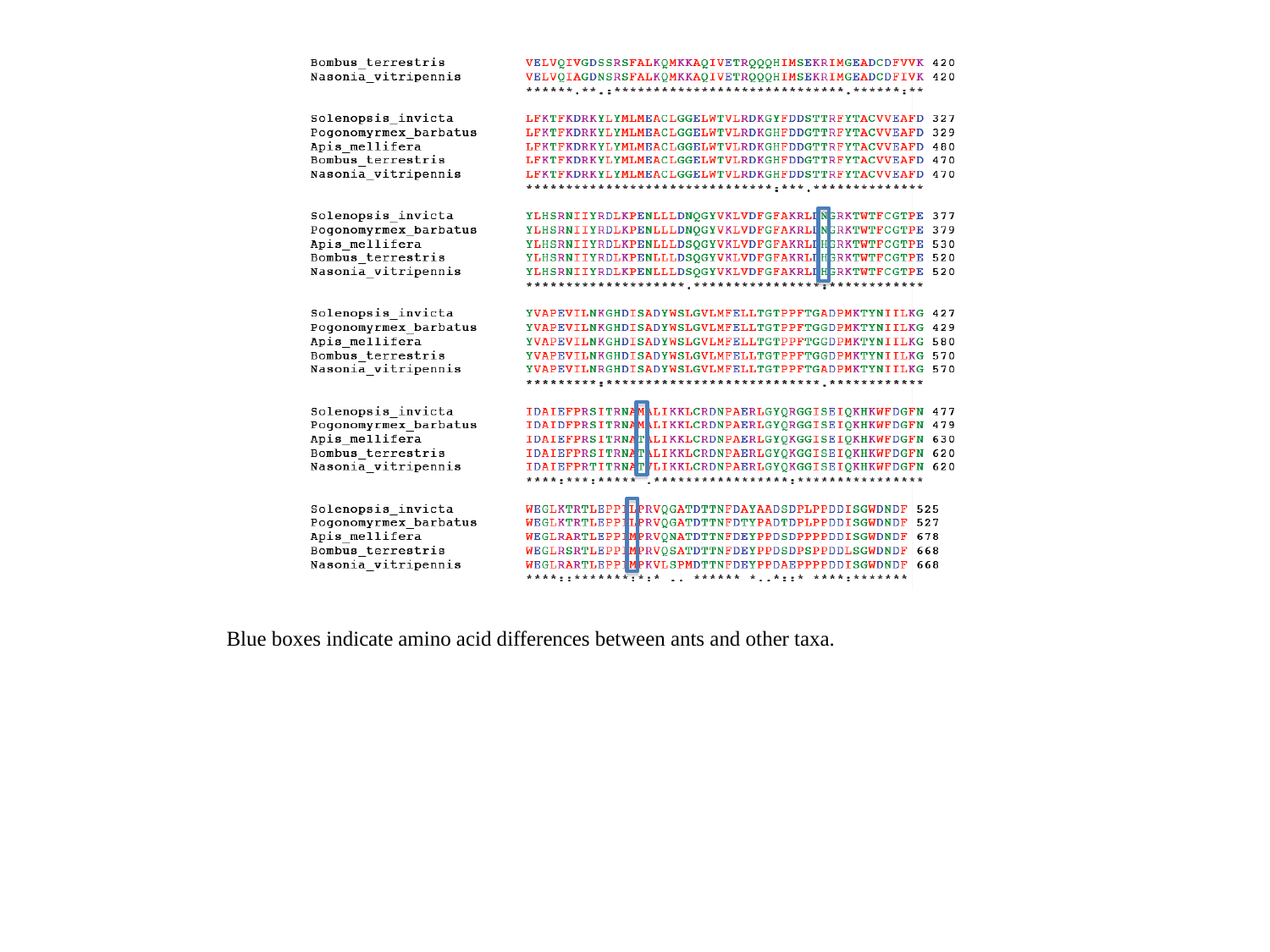

Blue boxes indicate amino acid differences between ants and other taxa.
