# Supplementary material for: Differential regulation of the foraging gene associated with task behaviors in harvester ants
Source: BMC Ecol. 2011 Aug 10;11:19. doi: 10.1186/1472-6785-11-19 (PMC3180247; doi:10.1186/1472-6785-11-19)
Supplement: Additional file 2 — Results from Mixed Model Repeated Measures ANOVA. Table of complete results from mixed model repeated measures ANOVA. [file 1472-6785-11-19-S2.DOC]

**Additional File #2:**

**Results from Mixed Model Repeated Measures ANOVA**

|  |  | **SS** | **df** | **MS** | **F** | **p** |
| --- | --- | --- | --- | --- | --- | --- |
| **Between Subjects** | |  | (21) |  |  |  |
|  | colony | 35.36 | 3 | 11.79 | 0.58 | NS |
|  | task | 2.23 | 1 | 2.23 | 0.11 | NS |
|  | colony X task | 73.56 | 3 | 24.52 | 1.21 | NS |
|  | subjects within | 325.45 | 8 | 40.68 |  |  |
|  |  |  |  |  |  |  |
| **Within Subjects** | |  | (120) |  |  |  |
|  | time | 66.54 | 3 | 22.18 | 5.33 | 0.001 |
|  | time X colony | 130.16 | 9 | 14.46 | 3.48 | 0.001 |
|  | time X task | 27.04 | 3 | 9.01 | 2.97 | 0.05 |
|  | time X colony X task | 420.00 | 27 | 15.56 | 11.21 | 0.001 |
|  | time X subjects within | 199.74 | 69 | 2.89 |  |  |
|  |  |  |  |  |  |  |
