# Supplementary material for: Differential regulation of the foraging gene associated with task behaviors in harvester ants
Source: BMC Ecol. 2011 Aug 10;11:19. doi: 10.1186/1472-6785-11-19 (PMC3180247; doi:10.1186/1472-6785-11-19)
Supplement: Additional file 3 — Database accession numbers for foraging gene. A list of the Genbank accession numbers for foraging gene sequences used in phylogenetic analyses. [file 1472-6785-11-19-S3.DOC]

Additional File #3:

Database accession numbers for *foraging* gene

| **Species** | **Accession numbers** |
| --- | --- |
| Acromyrmex echinatior | EGI70107.1 |
| Acyrthosiphon pisum | XP_001952091.1 |
| Anopheles gambiae | XP_319605.4 |
| Apis mellifera | NP_001011581.1 |
| Atta cephalotes | from genome database |
| Bombus ignitus | BAH29963.1 |
| Bombus terrestris | ACS36224.1 |
| Caenorhabditis elegans | O76360.2 |
| Camponotus floridanus | EFN63550.1 |
| Diabrotica virgifera virgifera | ABI97017.1 |
| Drosophila melanogaster | ACO44435.1 |
| Harpegnathos saltator | EFN87545.1 |
| Linepithema humile | from genome database |
| Locusta migratoria | ACV89934.1 |
| Mythimna separata | ACX46913.1 |
| Nasonia vitripennis | XP_001603549.1 |
| Pheidole pallidula | ABW22624.1 |
| Pogonomyrmex barbatus | AAV65146.1 |
| Pogonomyrmex occidentalis | JN255751 |
| Pristionchus pacificus | ACB59340.1 |
| Schistocerca gregaria | ACV89935.1 |
| Schistosoma japonicum | AAX26936.2 |
| Solenopsis invicta | EFZ12468.1 |
| Tribolium castaneum | XP_973707.2 |
| Vespula vulgaris | ABL74445.1 |
