# Supplementary material for: Differential regulation of the foraging gene associated with task behaviors in harvester ants
Source: BMC Ecol. 2011 Aug 10;11:19. doi: 10.1186/1472-6785-11-19 (PMC3180247; doi:10.1186/1472-6785-11-19)

## Slide 1
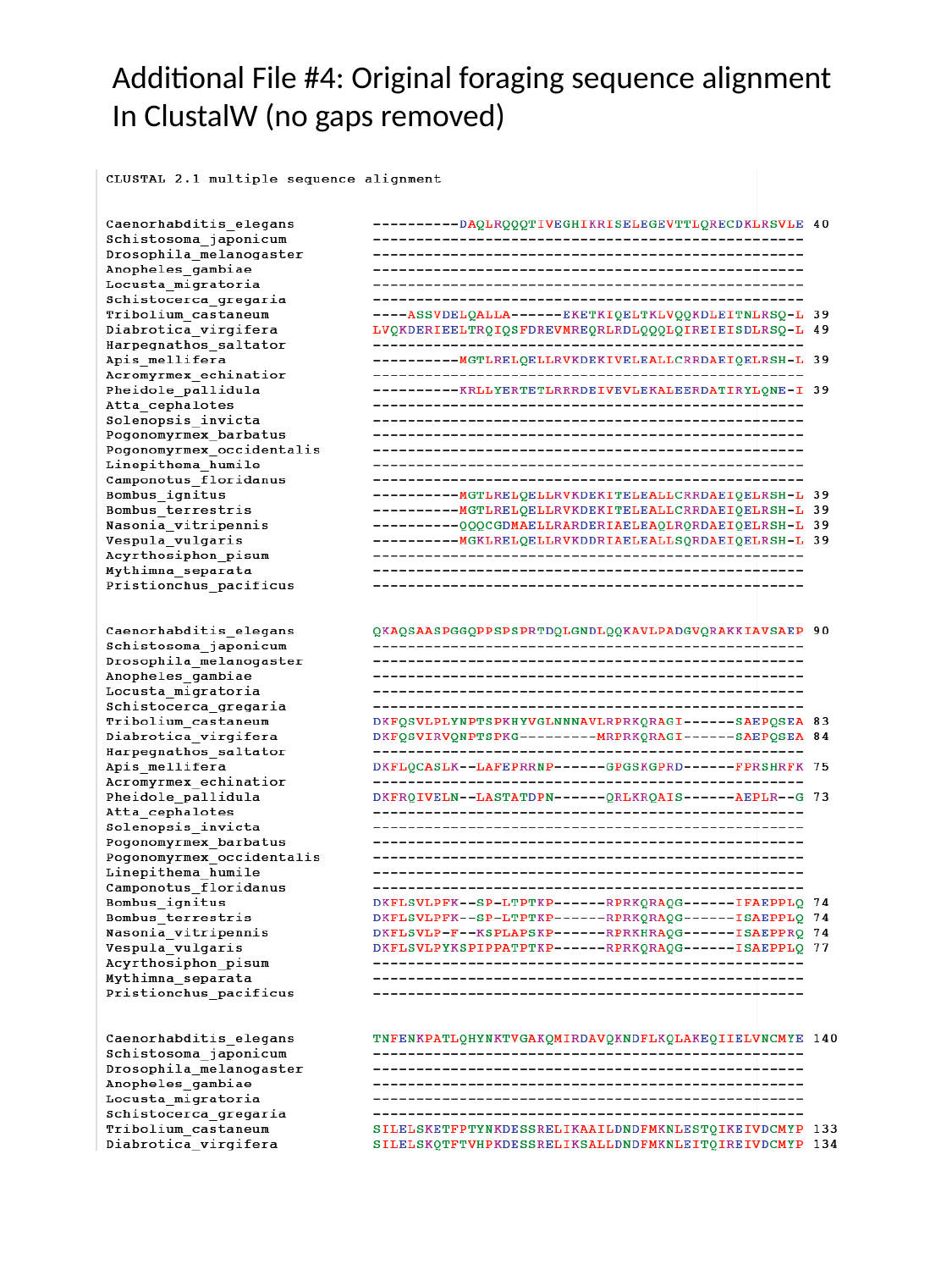

Additional File #4: Original foraging sequence alignment
In ClustalW (no gaps removed)

## Slide 2
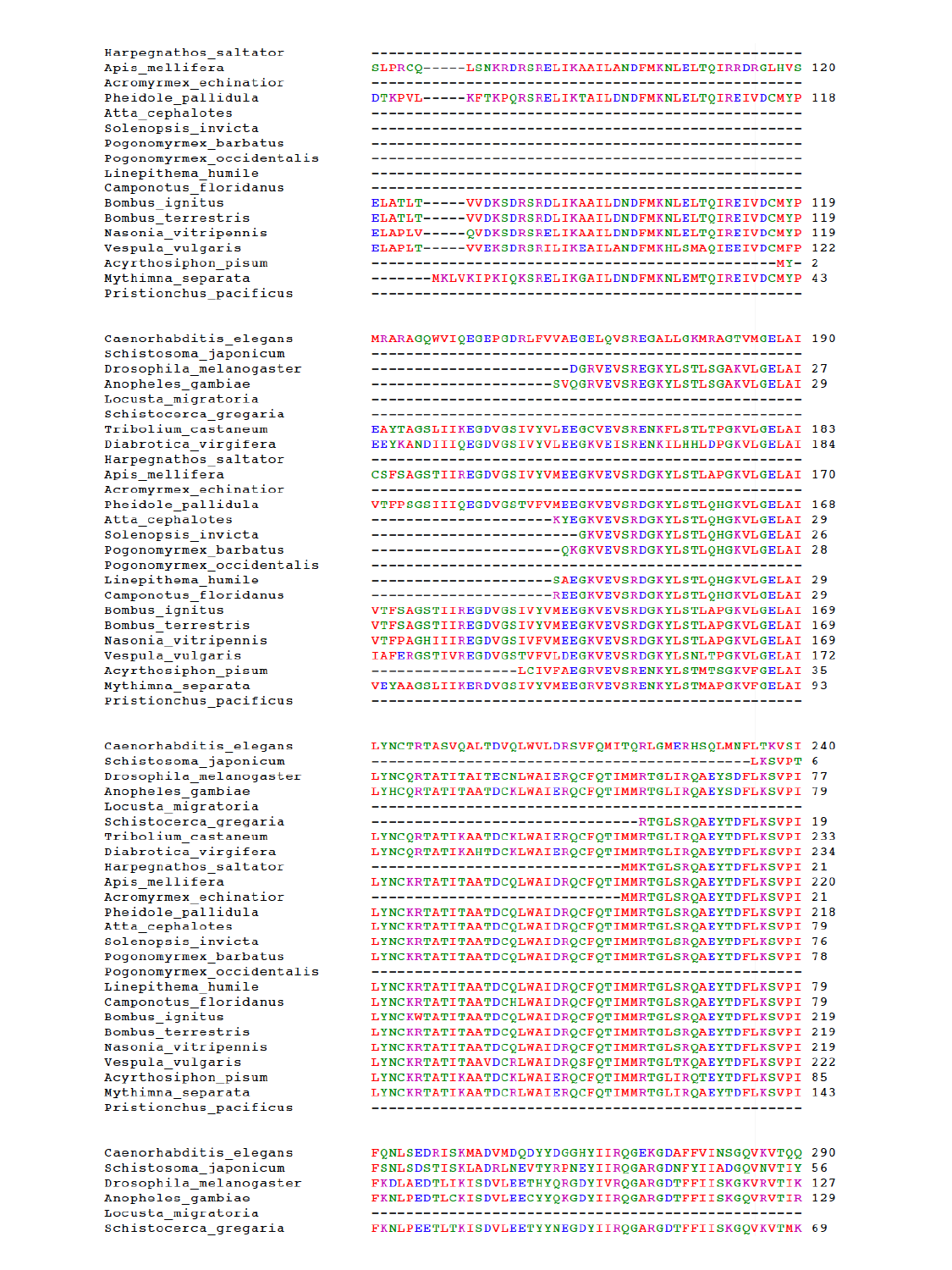

## Slide 3
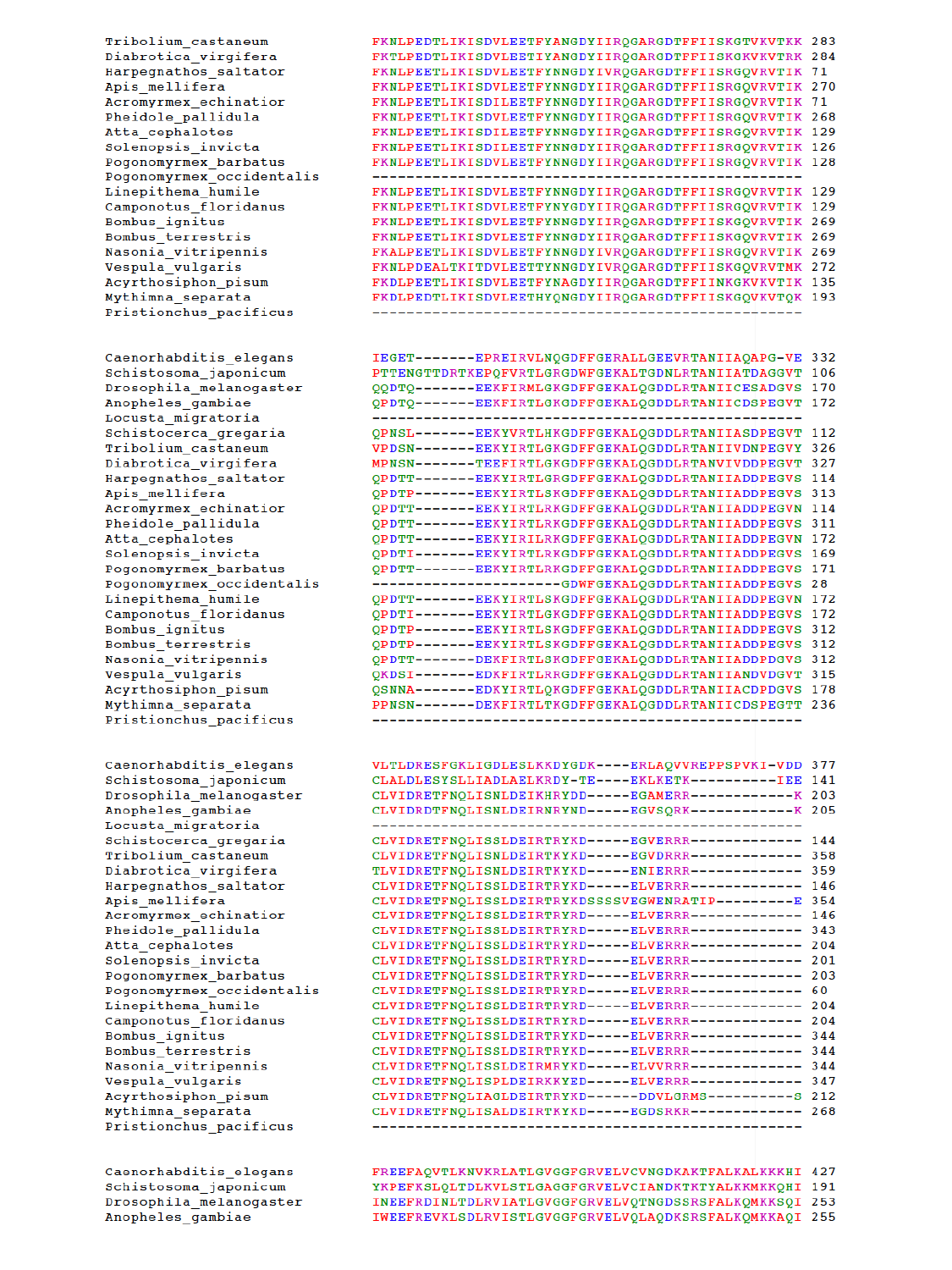

## Slide 4
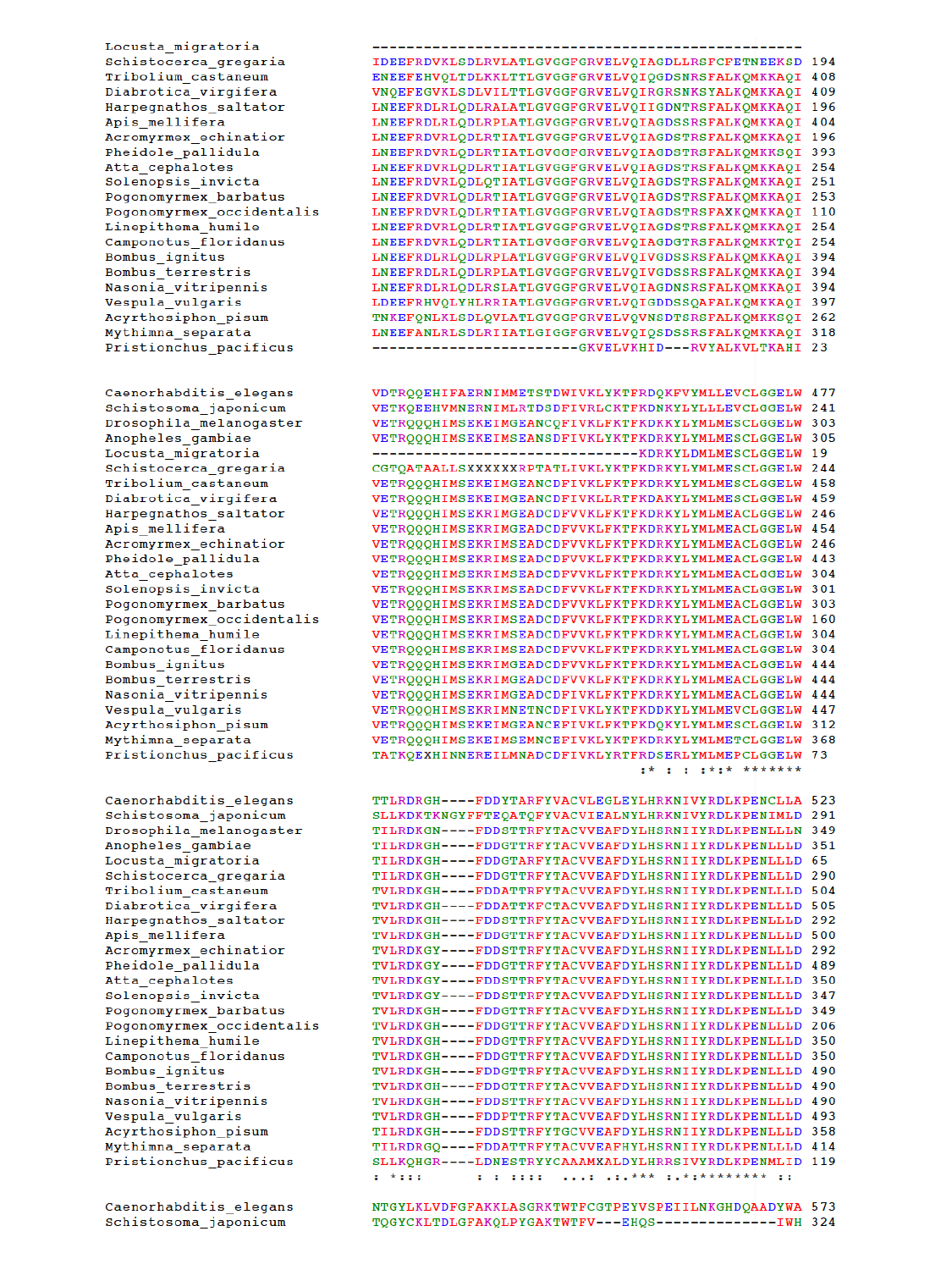

## Slide 5
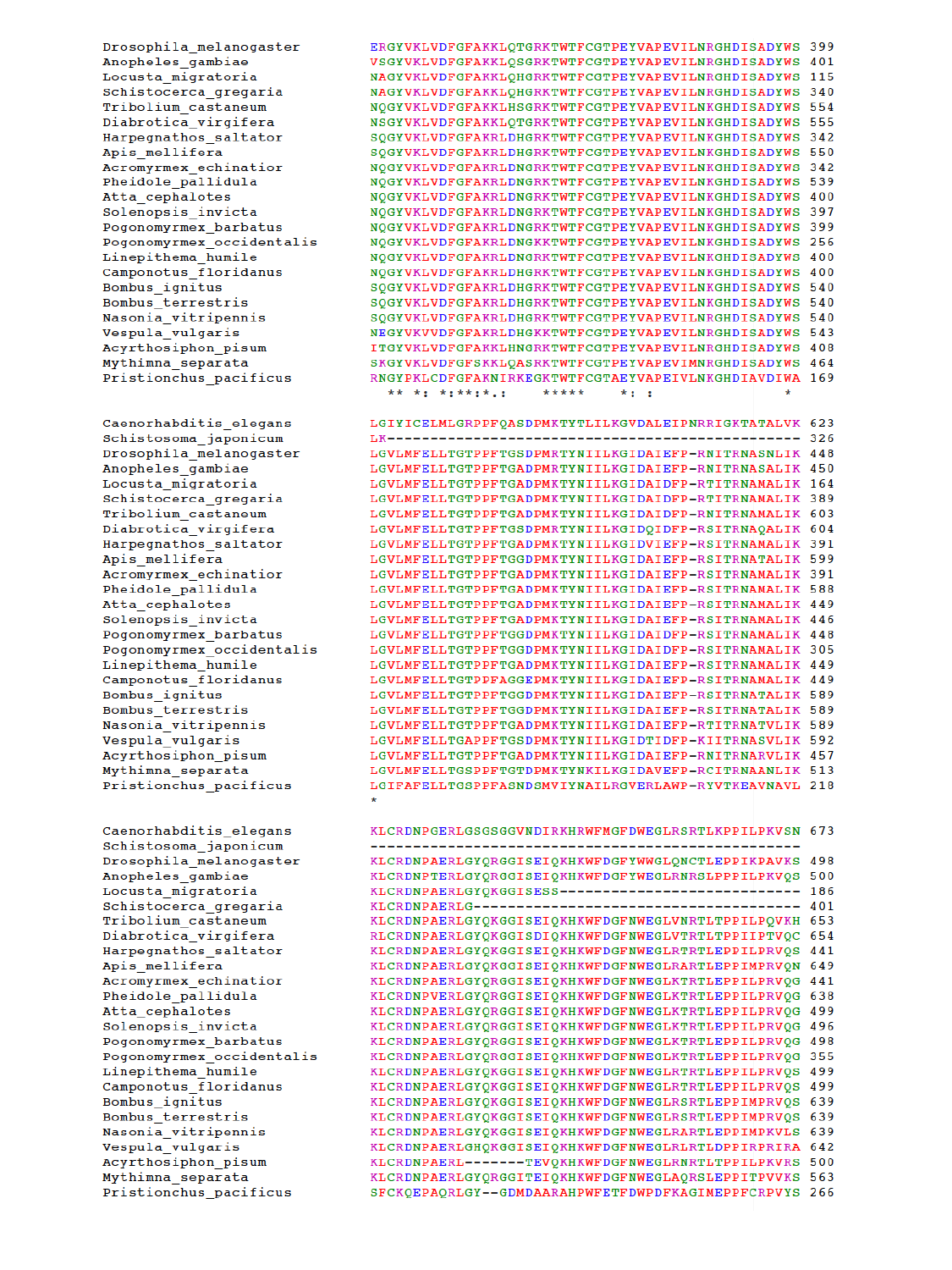

## Slide 6
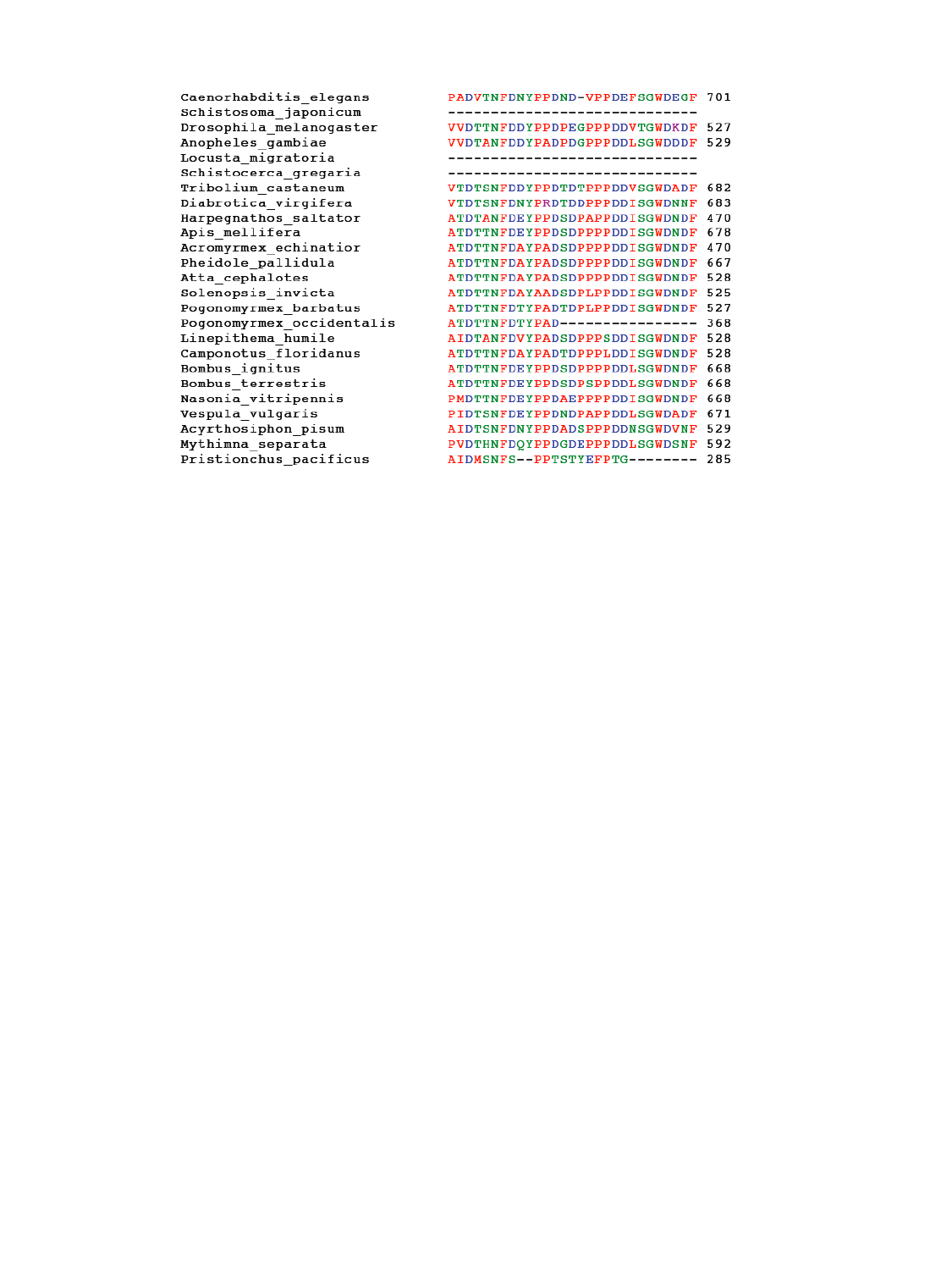

Supplement: Additional file 4 — Original foraging sequence alignment in ClustalW. Alignment of foraging gene sequences using Clustal W with no gaps removed. This alignment was used to measure similarity scores of amino acid seqeuences between species. [file 1472-6785-11-19-S4.PPT]
