# Supplementary material for: Differential regulation of the foraging gene associated with task behaviors in harvester ants
Source: BMC Ecol. 2011 Aug 10;11:19. doi: 10.1186/1472-6785-11-19 (PMC3180247; doi:10.1186/1472-6785-11-19)
Supplement: Additional file 5 — Maximum parsimony tree of foraging gene. Maximum parsimony consensus tree of foraging gene. The tree shown represents the bootstrap consensus tree of 250 replicates. [file 1472-6785-11-19-S5.PPT]

## Slide 1
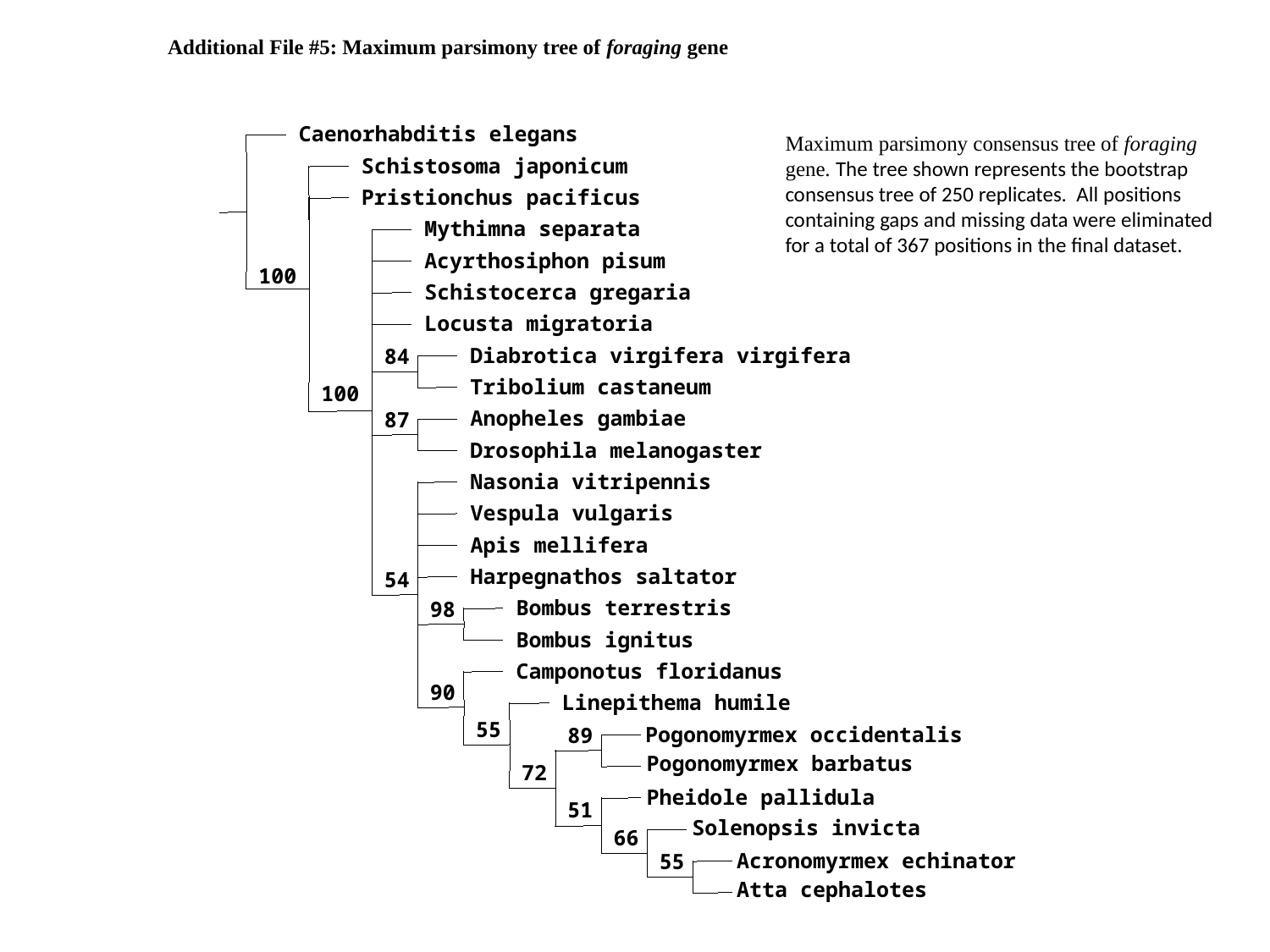

Additional File #5: Maximum parsimony tree of foraging gene
Caenorhabditis elegans
Schistosoma japonicum
Pristionchus pacificus
Mythimna separata
Acyrthosiphon pisum
100
Schistocerca gregaria
Locusta migratoria
Diabrotica virgifera virgifera
84
Tribolium castaneum
100
Anopheles gambiae
87
Drosophila melanogaster
Nasonia vitripennis
Vespula vulgaris
Apis mellifera
Harpegnathos saltator
54
Bombus terrestris
98
Bombus ignitus
Camponotus floridanus
90
Linepithema humile
55
Pogonomyrmex occidentalis
89
Pogonomyrmex barbatus
72
Pheidole pallidula
51
Solenopsis invicta
66
Acronomyrmex echinator
55
Atta cephalotes
Maximum parsimony consensus tree of foraging gene. The tree shown represents the bootstrap consensus tree of 250 replicates. All positions containing gaps and missing data were eliminated for a total of 367 positions in the final dataset.
